# Early NCCT imaging signs for prognostication in intracerebral hemorrhage: a retrospective cohort study with long follow up results

**DOI:** 10.1186/s12883-025-04100-z

**Published:** 2025-03-06

**Authors:** Rong Deng, Chuyue Wu, Lina Zhang, Jing Wang, Jing Guo, Zhenjie Yang, Lei He, Shengli Chen

**Affiliations:** 1 Department of Neurology, Chongqing University Three Gorges Hospital, No.165 Xincheng Road, Wanzhou District, Chongqing, 404100 China; 2 School of Medicine, Chongqing University, Chongqing, 404010 China; 3 NHC Key Laboratory of Diagnosis and Treatment on Brain Functional Diseases, The First Affiliated Hospital of Chongqing Medical University, Chongqing, 400016 China; 4 Chongqing Municipality Clinical Research Center for Geriatric Diseases, Chongqing University Three Gorges Hospital, Wanzhou, Chongqing, 404000 China; 5 Department of Radiology, Chongqing University Three Gorges Hospital, Wanzhou, Chongqing, 404000 China

**Keywords:** Intracerebral hemorrhage, Poor prognosis, NCCT signs, Prediction model, Stroke

## Abstract

**Objective:**

This study intends to investigate the connection between non-contrast computed tomography (NCCT) imaging findings and neurological function scores in patients with intracerebral hemorrhage (ICH) in a long follow up of 451 patients.

**Methods:**

Between January 2020 and October 2021, a retrospective review was undertaken on patients with ICH. The NCCT imaging results within 24 h of symptom onset, clinical information, biochemical markers and the one-year outcome post-discharge were collected and analyzed. Subsequently, a prognostic model was devised to predict poor outcomes.

**Results:**

A cohort of 451 patients diagnosed with ICH was analyzed in this study. Adverse prognostic outcomes at three months were found to be independently associated with several factors, including the presence of the swirl sign (*P* = 0.010), advanced age (*P* = 0.003), post-ICH modified Rankin Scale (mRS) score (*P* = 0.003,), time elapsed from symptom onset to NCCT scan (*P* = 0.018), and the presence of ventricular hemorrhage (*P* = 0.003). Unfavorable prognosis at 12 months was independently associated with the presence of the island sign (*P* = 0.001), older age (*P* = 0.003), post-ICH mRS score (*P* = 0.003), and HE (*P* = 0.014). Additionally, the integration of NCCT imaging signs into the predictive model significantly improved its accuracy in predicting adverse outcomes at both three months (AUC = 0.817 vs. 0.782 in the model without NCCT, NRI = 0.219, *P* = 0.033, IDI = 0.080, *P* = 0.006) and 12 months (AUC = 0.829 vs. 0.797 in the model without NCCT, NRI = 0.235, *P* = 0.028, IDI = 0.096, *P* = 0.003).

**Conclusions:**

The early imaging features of patients suffering from ICH can provide a more precise prognosis from the analysis of the 12-month follow up results.

**Supplementary Information:**

The online version contains supplementary material available at 10.1186/s12883-025-04100-z.

## Introduction

Intracerebral hemorrhage (ICH) is a critical medical condition characterized by the spontaneous rupture of blood vessels in the brain parenchyma, resulting in blood accumulation [1]. With an incidence rate of 12–15 cases per 100,000 individuals every year, ICH significantly burdens global healthcare systems. The prognosis of ICH is poor, with a high case fatality rate of 54% within one year of onset, underscoring the pressing need for effective interventions [1, 2]. Long-term functional independence is challenging for ICH survivors, with only a minority (12% to 39%) achieving it. This challenge is exacerbated in cases of anticoagulation-associated ICH, where up to 76% of patients experience mortality or permanent loss of functional independence [3].

The complexity of ICH outcomes is influenced by various factors such as hematoma volume, location, extension to ventricles, and other variables, highlighting the multifaceted nature of the condition [4, 5]. Despite the critical role of prognostic prediction in guiding personalized interventions and enhancing outcomes for ICH patients, the existing prognostic tools are inadequate. There is an urgent need for robust and accurate methods to predict individual prognoses and customize treatment strategies accordingly. The absence of such tools limits clinicians' ability to deliver timely and tailored interventions, thereby hindering optimal care for ICH patients.

The persistence of early bleeding events in ICH can contribute to hematoma expansion (HE), and is related with death and disability [6]. Therefore, timely identification and prediction of ICH are essential for guiding treatment decisions and enhancing patient outcomes. Several computed tomography (CT) imaging signs, visible within 24 h of onset, have been established as dependable predictors of ICH [7, 8]. Various signs of HE on CT scans include features such as heterogeneous density, irregular hematoma shapes, satellite, swirl, black hole, and blend characteristics [8]. The accuracy of these CT signs in predicting ICH has been validated across multiple medical centers, underscoring their clinical utility and reproducibility. Recent studies have also elucidated the prognostic implications of CT signs, underscoring their significance in clinical management and patient outcomes [8–10]. The integration of these imaging biomarkers into prognostic models holds promise in refining risk stratification precision and tailoring personalized interventions for patients with ICH resulting in a model that may be helpful in clinical practice.

Our study aimed to develop prognosis prediction models to improve personalized interventions for individuals with ICH from the long follow up results. We sought to assess the value of non-contrast computed tomography (NCCT) imaging in informing these models and gaining insights into ICH management. Our multifaceted approach incorporated features like NCCT imaging findings, biochemical properties, clinical indicators, and medical histories. By integrating these variables, we devised an innovative modeling strategy to predict ICH patient prognosis more accurately. This comprehensive approach shows promise in advancing prognostic assessment and enabling tailored interventions in ICH clinical management.

## Method

### Study design

The study involved a retrospective analysis of participants who were prospectively enrolled in an ongoing cohort at the Advanced Stroke Center of Chongqing University Three Gorges Hospital. Approval for all study protocols was obtained from the Clinical Trial Ethics Committee of Chongqing University Three Gorges Hospital (No. 20220042), and informed consent was acquired from all subjects or their surrogates in adherence to the Declaration of Helsinki. Additionally, the study was registered in the National Medical Research Registration and Archival Information System (the unique identifier: MR-50–23–001346).

### Participants

**Fig. 1:**
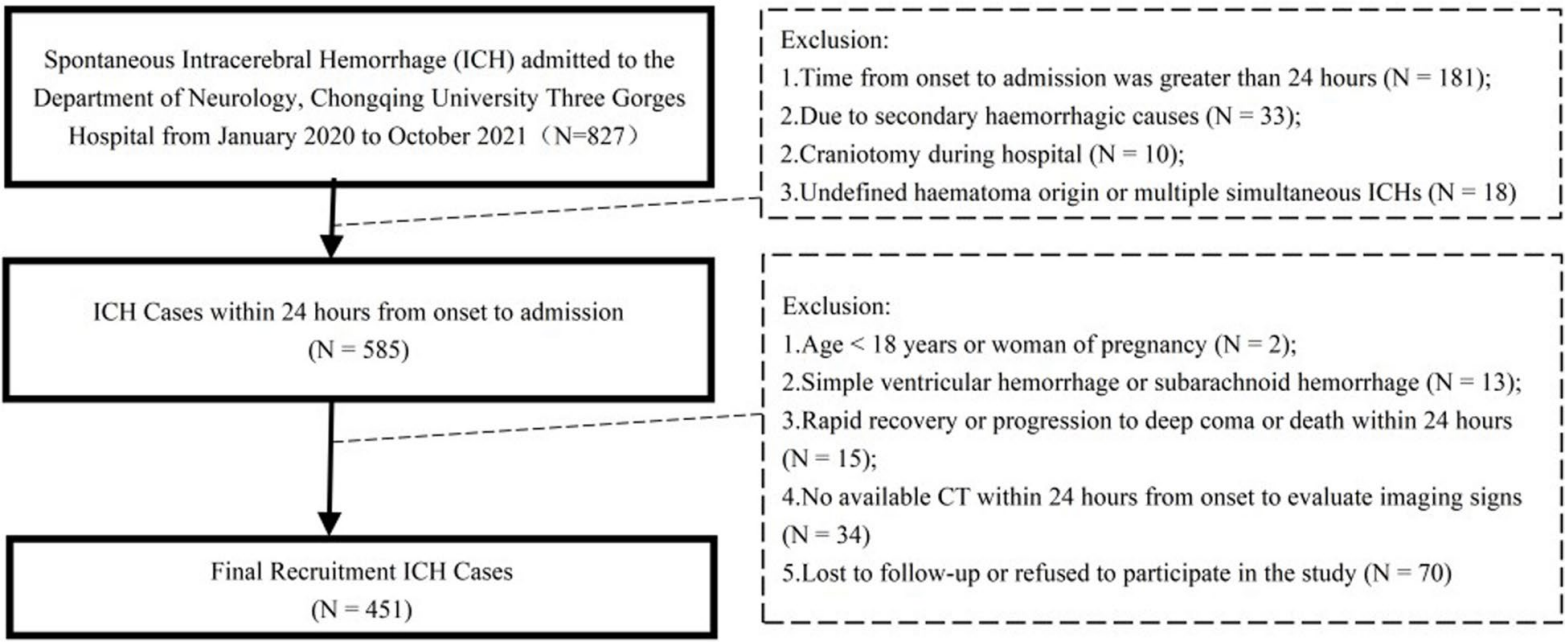
Flow diagram of eligibility of ICH Cases

The study included patients admitted to the Advanced Stroke Center within the Department of Neurology at Chongqing University Three Gorges Hospital from January 2020 to October 2021. The inclusion criteria were: 1) individuals over 18 years old and not pregnant; 2) meeting the diagnostic criteria for spontaneous ICH as outlined in the 2019 Chinese Guidelines for the Diagnosis and Treatment of ICH; 3) admission within 24 h of symptom onset; and 4) availability of a NCCT scan within 24 h of symptom onset to assess imaging signs. The exclusion criteria included: 1) secondary cerebral hemorrhage-related factors such as tumor, trauma, abnormal vascular structure, cerebral infarction hemorrhage transformation; 2) simple ventricular hemorrhage or subarachnoid hemorrhage; 3) undefined hematoma origin; 4) craniotomy during hospitalization; 5) progression quickly to death within 24 h after admission; and 6) loss to follow-up or refusal to participate in the study. The eligibility of ICH cases is depicted in Fig. 1 through a flow diagram.

### Clinical data

Data extracted from medical records included demographic details (gender, age), medical history (hypertension, diabetes, coronary heart disease, atrial fibrillation, use of anticoagulant/antiplatelet medications, prior stroke, smoking, alcohol consumption), admission specifics (systolic and diastolic blood pressure, pre- and post-stroke modified Rankin Scale (mRS) scores [11], Glasgow Coma Scale (GCS) score [12], National Institutes of Health Stroke Scale (NIHSS) score) [13], hematoma characteristics (location—lobar, deep, cerebellar, or brainstem; volume), time from symptom onset to initial NCCT scan, presence of ventricular and/or subarachnoid hemorrhage, midline shift, SMASH-U classification (Structural vascular lesions, Medication, Amyloid angiopathy, Systemic disease, Hypertension, Undetermined), and serological markers (Total Cholesterol (TC), Triglycerides (TG), High-density Lipoprotein (HDL), Low-density Lipoprotein (LDL), C-reactive protein (CRP), Creatinine (CR), Glomerular Filtration Rate (GFR), Platelet count (PLT), Alanine Aminotransferase (ALT), Aspartate Aminotransferase (AST), Glycated Hemoglobin (GHB), Prothrombin Time International Normalized Ratio (PT-INR), Fibrinogen (FIB), all obtained within one hour of admission.

### Imaging evaluation

Classification of hemorrhage locations included lobar, deep, cerebellar, and brainstem regions [14], with consideration of hemorrhage extension into the ventricle [15] and subarachnoid space [16]. Hematoma volume was determined using the ellipsoid formula (A × B × C/2) in mL, and analysis of midline shift and hematoma orientation was conducted based on established methodologies [17, 18].

### Hematoma expansion (HE)

Both the first CT scan (CT1) and the follow-up CT scan (CT2) need to be performed within 24 h of the onset of ICH. Hematoma enlargement, defined by the INTERACT2 criteria [19], is indicated by a > 33% or > 12.5 ml expansion in volume on CT2 relative to CT1.

### Heterogeneous density and irregular shape

Barras et al. [20] proposed an assessment method using a 5-point scale to measure shape irregularity and density heterogeneity, with ratings spanning from 1 (representing high regularity and evenness) to 5 (representing significant irregularity and unevenness). Cases where the score surpassed 2 points were noted for exhibiting heterogeneous density and irregular shape [19].

### Island sign

The island sign is characterized by either three or more small hematomas detached from the main hematoma or four or more small hematomas partially or completely separated from it [21]. These dispersed small hematomas, referred to as island hematomas, display a rounded or oval shape and are distinguishable from the primary hematoma. It is essential to observe that small hematomas linked to the primary hematoma should exhibit a vesicular or sprouted appearance rather than a lobulated one.

### Satellite sign

The satellite sign is defined by the complete detachment of a minor hematoma from the principal hematoma on a solitary layer of a NCCT scan [20]. The minor hematoma typically measures < 10 mm in diameter and is 1–20 mm away from the primary hematoma. Differentiating satellite signs from intraventricular hemorrhage and subarachnoid hemorrhage is crucial.

### Swirl sign

The swirl sign manifests as regions of low or equal density within high-density areas across two successive CT image layers in comparison to brain parenchymal density [22]. These regions can present in diverse shapes, including round, striped, or irregular.

### Black hole sign

The black hole sign refers to a hypodense region identified on CT scans, enclosed by a hematoma with distinct borders [13]. The CT attenuation value of the adjacent brain tissue is ≥ 28 Hounsfield units (HU).

### Blend sign

The blend sign is characterized by a juxtaposition of low-density and high-density areas within the hematoma, separated by a distinct boundary and a density difference of at least 18 HU between the 2 density regions [23]. Crucially, the low-density region must not be entirely enclosed by the high-density region. Two neuroradiological specialists independently assessed all imaging studies without knowing the clinical details. Discrepancies were resolved by involving a third expert for a final adjudication.

### Patient follow-up

Follow-up were done through telephone interviews, and outpatient appointments with the participants. The follow up data was obtained from the outpatient interview using the required scales. The follow-up duration commenced at discharge and continued until death or 12 months post-onset. Statistical analysis encompassed mRS scores at 3 and 12 months, with a mRS score below 3 indicating a favorable prognosis, while any other score or mortality denoted a poor prognosis.

### Statistical analysis

Categorical variables were presented as frequencies and percentages and assessed using the likelihood-ratio chi-squared test. Continuous variables were expressed as either the mean ± standard deviation or median and interquartile range (IQR), with group comparisons conducted using either the Mann‒Whitney U test or T-test, depending on normality assumptions.

Unconditional logistic regression models were employed to evaluate the impact of NCCT imaging on the prognosis of patients with ICH at 3 or 12 months, in conjunction with other relevant factors. The results presented include P values, odds ratios (ORs), 95% confidence intervals (CIs) for categorical variables, and P values, T scores, or U scores for continuous variables. A predictive model for poor prognosis was constructed through multivariate logistic regression analysis, integrating pertinent indicators. Further analyses comprised the receiver operating characteristic (ROC) curve, the area under the ROC curve (AUC), sensitivity, and specificity. The net reclassification index (NRI) and integrated discrimination improvement (IDI) were utilized to quantify enhancements in model performance. The NRI assesses the predictive ability of new and old models at a specified cutoff value, while the IDI evaluates prediction accuracy between old and new models [24]. Statistical analyses and graphical representations were performed using R version 4.0.4 and SPSS software version 22.

## Results

### Patients’ basic characteristics

A cohort of 451 patients diagnosed with ICH was examined, consisting of 280 males (62.1%) and 171 females (37.9%), with a mean age of 59.24 ± 12.08 years (median age 58, IQR 52–68). Within the cohort, 349 patients (77.4%) had a history of hypertension, 41 patients (9.1%) had diabetes, 23 patients (5.1%) had ischemic heart disease, five patients (1.1%) had atrial fibrillation, 15 patients (3.3%) had a prior history of anticoagulant/antiplatelet drug use, 77 patients (17.1%) had a history of stroke, 159 patients (35.3%) were smokers, and 164 patients (36.4%) reported alcohol consumption. The etiological SMASH-U classification revealed 28 cases of structural vascular lesions (6.2%), 12 cases attributed to medication (2.7%), 36 cases of amyloid angiopathy (8.0%), 16 cases related to systemic diseases (3.5%), and 359 cases associated with hypertension (79.6%). Regarding treatment, 228 patients (50.6%) received solely medical therapy, while 223 (49.4%) underwent minimally invasive surgery.

The study found an average hematoma volume of 22.80 ± 20.00 ml and a mean time interval between symptom onset and initial NCCT scan of 3.56 ± 3.18 h. Lobar hemorrhage was present in 110 cases (24.4%), deep hemorrhage in 293 cases (65.0%), cerebellar hemorrhage in 18 cases (4.0%), and brainstem hemorrhage in 30 cases (6.7%). Additionally, ventricular hemorrhage was observed in 178 cases (39.5%), subarachnoid hemorrhage in 96 cases (21.3%), and midline displacement in 376 cases (83.4%), with 204 cases (54.3%) exhibiting left shift and 172 cases (45.7%) showing right shift.

The median mRS score before ICH was 0 (IQR = 0), while post-ICH, it stood at 4 (IQR = 1). Upon admission, patients presented with a median GCS score of 13 (IQR = 6) and a median NIHSS score of 14 (IQR = 12). At the 3-month mark, the median mRS score was 2 (IQR = 3), indicating that 214 patients (47.5%) had an unfavorable prognosis, whereas 237 patients (52.5%) had a favorable prognosis. By the 12-month follow-up, the median mRS score remained at 2 (IQR = 3), with 199 patients (44.1%) demonstrating a poor prognosis and 252 patients (55.9%) exhibiting a good prognosis.

### Correlation of NCCT imaging with prognosis

**Fig. 2:**
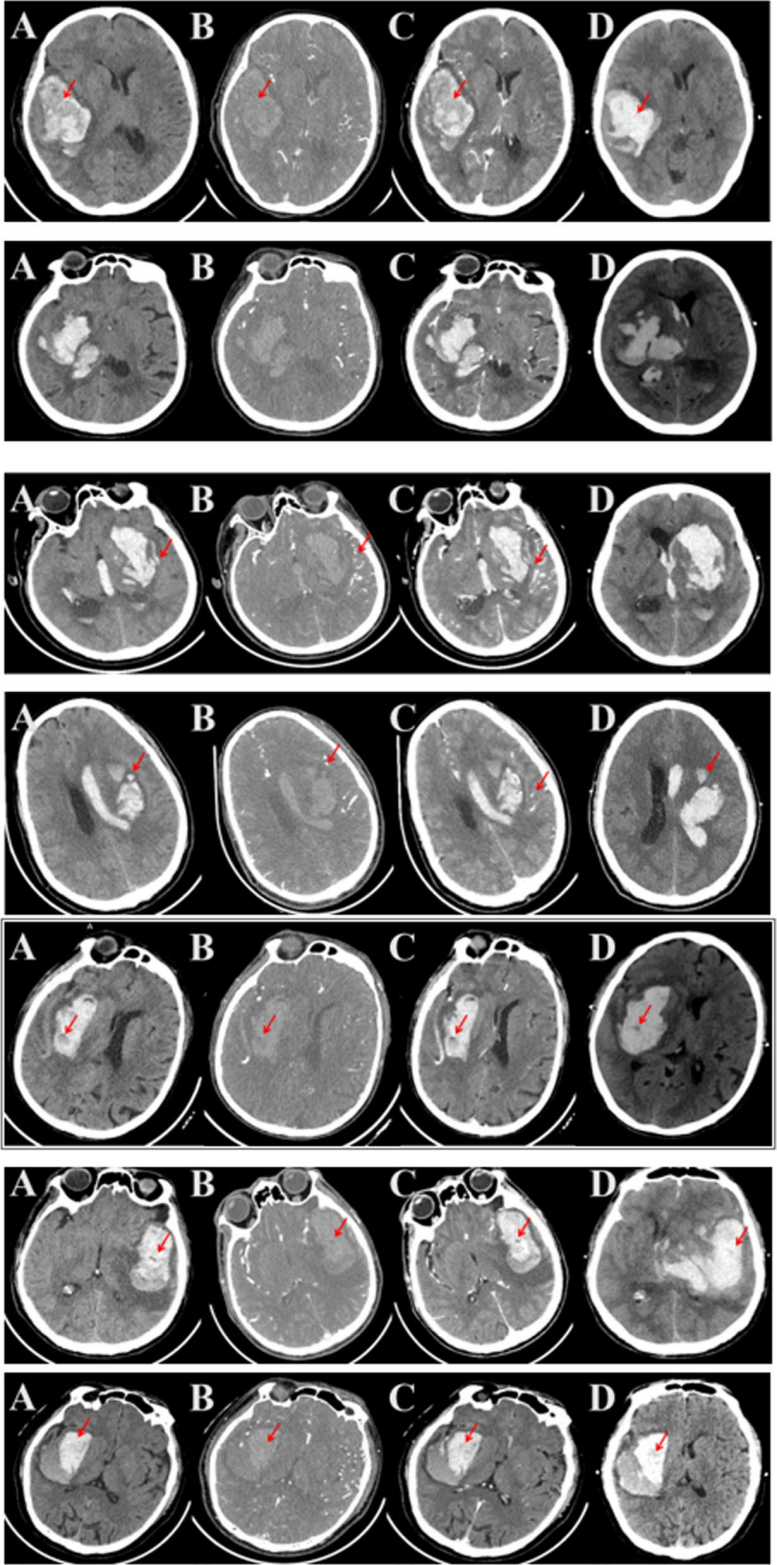
Representative imaging pictures of CT scan. From top to bottom was Homogenous density, Irregular shape, Island sign, Satellite sign, Swirl sign, Black hole sign, Blend sign. **A** Plain CT scan; **B** CT enhancement; **C** Sequential CT plain scan; **D** Reexamination CT scan in 24 h. The arrow represented the signs position

A total of 62 patients (16.9%) presented with hematoma expansion (HE) on a follow-up NCCT scan. Among the observed NCCT signs, the following frequencies were noted: "heterogeneous density" in 168 cases (37.3%), "irregular shape" in 216 cases (47.9%), "island sign" in 95 cases (21.1%), "satellite sign" in 117 cases (25.9%), "swirl sign" in 167 cases (37.0%), "black hole sign" in 50 cases (11.1%), and "blend sign" in 87 cases (19.3%). The distribution of NCCT signs within 24 h post-onset between patients with poor and good prognoses is provided in Table 1. The representative imaging pictures of NCCT scan were shown in Fig. 2.

**Table 1:** The comparison of CT sign frequency within the first 24 h post-onset among groups with poor and good prognoses

Prognosis at 3 months	Cases (*N* = 451) N	Poor prognosis (*N* = 214) N(%)	Good prognosis(*N* = 237) N(%)	P	OR	95% CI
Heterogeneous density	168	87(40.7)	81(34.2)	0.155	1.319	0.900,1.934
Irregular shape	216	119(55.6)	97(40.9)	**0.002**	1.808	1.244,2.627
Island sign	95	58(27.1)	37(15.6)	**0.003**	2.010	1.266,3.191
Satellite sign	117	62(29.0)	55(23.2)	0.163	1.350	0.885,2.059
Swirl sign	167	92(43.0)	75(31.6)	**0.013**	1.629	1.108,2.394
Black hole sign	50	34(15.9)	16(6.8)	**0.002**	2.609	1.395,4.879
Blend sign	87	47(22.0)	40(16.9)	0.172	1.386	0.867,2.216
Prognosis at 12 months	Cases (*N* = 451) N	Poor prognosis(*N* = 199) N(%)	Good prognosis(*N* = 252) N(%)	P	OR	95% CI
Heterogeneous density	168	79(39.7)	89(35.3)	0.339	1.206	0.821,1.770
Irregular shape	216	105(52.8)	111(44.0)	0.066	1.419	0.977,2.061
Island sign	95	56(28.1)	39(15.5)	**0.001**	2.139	1.350,3.389
Satellite sign	117	59(29.6)	58(23.0)	0.111	1.410	0.924,2.151
Swirl sign	167	82(41.2)	85(33.7)	0.103	1.377	0.937,2.023
Black hole sign	50	29(14.6)	21(8.3)	**0.036**	1.876	1.034,3.404
Blend sign	87	45(22.6)	42(16.7)	0.112	1.461	0.914,2.336

A good prognosis was defined as an mRS score lower than 3, whereas a poor prognosis, including death (score of 6), was indicated by a score of 3 or higher

At the 3-month prognosis assessment, 214 cases showed poor prognosis while 237 cases exhibited good prognosis. Patients with poor prognosis had a higher prevalence of "Irregular shapes" (119; 55.6% vs. 97; 40.9%, *P* = 0.002, OR = 1.808, 95% CI [1.244, 2.627]), "Island signs" (58; 27.1% vs. 37; 15.6%, *P* = 0.003, OR = 2.010, 95% CI [1.266, 3.191]), "Swirl signs" (92; 43.0% vs. 75; 31.6%, *P* = 0.013, OR = 1.629, 95% CI [1.108, 2.394]), and "Black hole signs" (34; 15.9% vs. 16; 6.8%, *P* = 0.002, OR = 2.609, 95% CI [1.395, 4.879]).

At the 12-month prognosis assessment, 199 cases exhibited a poor prognosis, while 252 cases showed a good prognosis. Notably, individuals with a poor prognosis demonstrated a higher prevalence of "Island signs" (56 cases, 28.1% vs. 39 cases, 15.5%; *P* = 0.001, OR = 2.139, 95% CI 1.350–3.389) and "Black hole signs" (29 cases, 14.6% vs. 21 cases, 8.3%; *P* = 0.036, OR = 1.876, 95% CI 1.034–3.404).

### Multivariate analysis of NCCT signs and other clinical data with poor prognosis

The characteristics of patient groups with favorable and unfavorable prognoses at 3 and 12 months post-onset are detailed in Supplemental Table 1 and Supplemental Table 2, respectively. The multivariate analysis in Table 2 reveals significant associations between NCCT imaging findings or other clinical parameters and poor prognostic outcomes. Specifically, at 3-months post-onset, a poor prognosis was independently linked to the presence of the swirl sign (*P* = 0.010, OR = 2.198, 95% CI (1.827,3.069)), advanced age (*P* = 0.003, OR = 1.042, 95% CI (1.024,1.061)), mRS score post-ICH (*P* = 0.003, OR = 1.499, 95% CI (1.212,1.853)), time elapsed from symptom onset to NCCT imaging (*P* = 0.018, OR = 0.994, 95% CI (0.989,0.999)), and the presence of ventricular hemorrhage (*P* = 0.003, OR = 2.422, 95% CI (2.005,3.386)). Similarly, at the 12-month mark, a poor prognosis was independently associated with the presence of the island sign (*P* = 0.001, OR = 2.666, 95% CI (2.389,3.670)), older age (*P* = 0.003, OR = 1.043, 95% CI (1.020,1.067)), mRS score post-ICH (*P* = 0.003, OR = 1.524, 95% CI (1.158,2.006)), and HE (*P* = 0.014, OR = 3.572, 95% CI (3.383,4.402)).

**Table 2:** The multivariate analysis regarding the correlation between CT signs or other clinical data and poor prognosis at 3 or 12 months post-onset

Risk factors	Prognostic indicators at 3-months after onset				
All cases (*N* = 451)	Poor Prognosis(*N* = 214) N(%) or Median(IQR)	Good prognosis(*N* = 237) N(%) or Median(IQR)	P	OR	95% CI
Swirl sign	92(43.0)	75(31.6)	0.010	2.198	1.827,3.069
Age, years	62.28 ± 11.95	56.50 ± 11.55	0.003	1.042	1.024,1.061
mRS score after ICH	5(1)	4(2)	0.003	1.499	1.212,1.853
Time from CT to onset, hours	2.86 ± 3.07	4.19 ± 3.50	0.018	0.994	0.989,0.999
Ventricular hemorrhage	100(46.7)	78(32.9)	0.003	2.422	2.005,3.386
Risk factors	Prognostic indicators at 1 year after onset				
All cases (*N* = 451)	Poor Prognosis(*N* = 199) N(%) or Median(IQR)	Good prognosis(*N* = 252) N(%) or Median(IQR)	P	OR	95% CI
Island sign	56(28.1)	39(15.5)	0.001	2.666	2.389,3.670
Age, years	62.40 ± 11.87	56.75 ± 11.68	0.003	1.043	1.020,1.067
mRS score after ICH	5(1)	4(2)	0.003	1.524	1.158,2.006
Hematoma expansion	42(25.8)	20(9.9)	0.014	3.572	3.383,4.402

A good prognosis was defined as an mRS score lower than 3, whereas a poor prognosis, including death (score of 6), was indicated by a score of 3 or higher

### Role of NCCT imaging in the prediction model

**Fig. 3:**
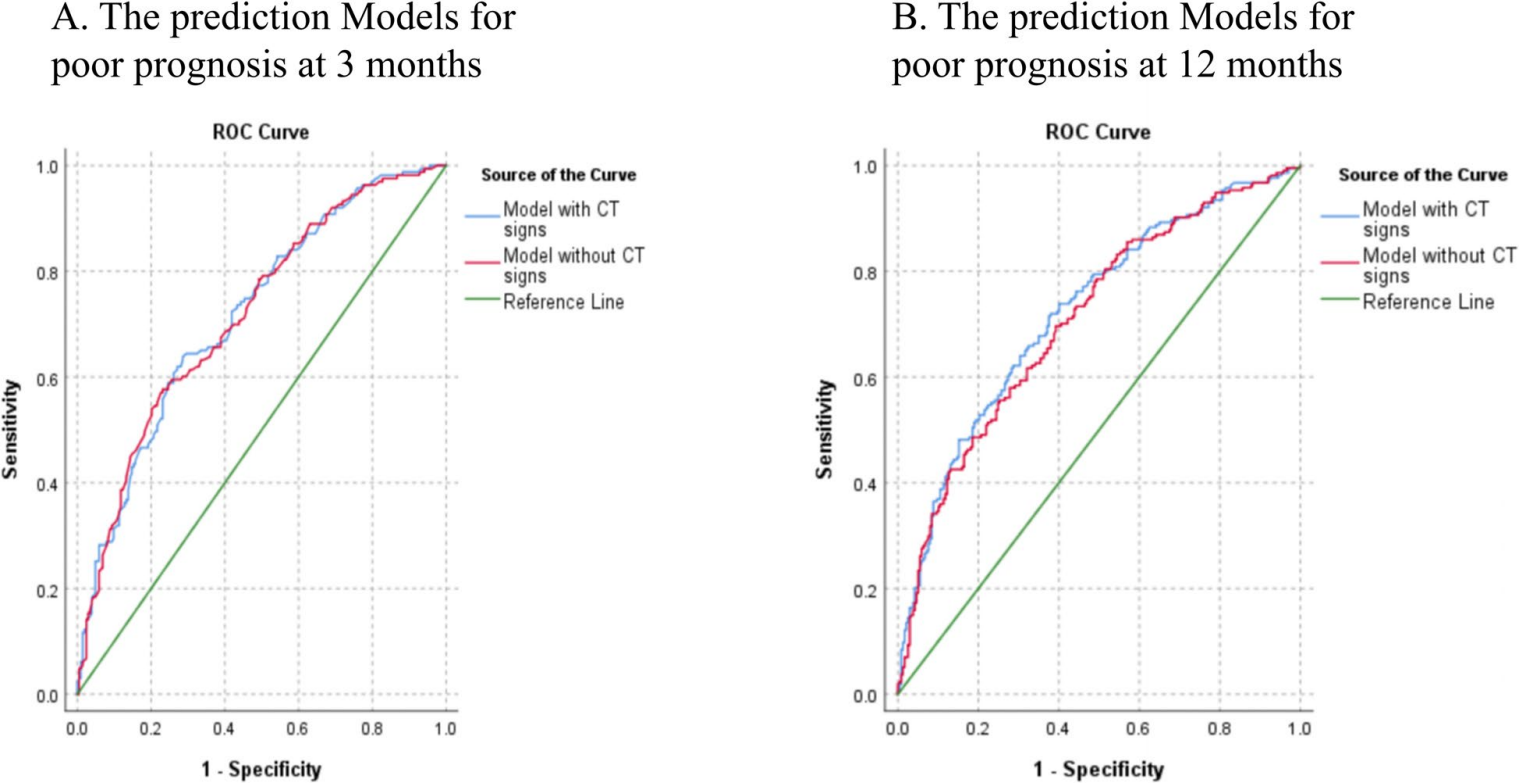
The ROC curves of prediction Models for poor prognosis at 3 and 12 months post-onset A good prognosis was defined as an mRS score lower than 3, whereas a poor prognosis, including death (score of 6), was indicated by a score of 3 or higher

The study compared the predictive performance of models with and without NCCT for 3-month and 12-month prognoses, as detailed in Table 3 and Fig. 3. The model incorporating NCCT exhibited superior performance for the 3-month prognosis, with an area under the curve (AUC) of 0.817, sensitivity of 0.720, and specificity of 0.620, compared to the model without NCCT (AUC = 0.782, sensitivity = 0.696, specificity = 0.608). The net reclassification improvement (NRI) (value = 0.219, *P* = 0.033, 95% CI 0.109–0.323) and integrated discrimination improvement (IDI) (value = 0.080, *P* = 0.006, 95% CI 0.023–0.127) demonstrated statistically significant differences between the two models for the 3-month prognosis. For the 12-month prognosis, the AUC of the model with NCCT (AUC = 0.829, sensitivity = 0.638, specificity = 0.714) surpassed that of the model without NCCT (AUC = 0.797, sensitivity = 0.577, specificity = 0.768). The NRI (value = 0.235, *P* = 0.028, 95% CI 0.063–0.408) and IDI (value = 0.096, *P* = 0.003, 95% CI 0.058–0.182) indicated significant disparities between the two models.

**Table 3:** Comparison of prediction efficiency indicators between the Model without CT and the Model with CT

Poor prognosis	Model without CT			Model with CT			NRI			IDI		
	AUC	Sensitivity	Specificity	AUC	Sensitivity	Specificity	Value	95% CI	P	Value	95% CI	P
3-months	0.782	0.696	0.608	0.817	0.720	0.620	0.219	0.109, 0.323	0.033	0.080	0.023, 0.127	0.006
12-months	0.797	0.577	0.768	0.829	0.638	0.714	0.235	0.063, 0.408	0.028	0.096	0.058, 0.182	0.003

A good prognosis was defined as an mRS score lower than 3, whereas a poor prognosis, including death (score of 6), was indicated by a score of 3 or higher

## Discussion

The poor prognosis at 12 months was independently associated with the island sign, older age, post-ICH mRS score, and HE. The integration of NCCT imaging signs into the predictive model significantly improved its accuracy in predicting adverse outcomes at 12 months.

The presence of the island sign, marked by multiple small bleeding sites encircling the primary hematoma, is a strong indicator of poor prognosis at 90 days for patients suffering from ICH [21]. Additionally, Zhang F et al. identified the island sign as an independent risk factor for unfavorable prognosis at one year in cases of ICH [25]. The island sign indicates the potential for multiple active hemorrhages, which can trigger thrombin production and initiate an inflammatory response. This inflammation may lead to blood–brain barrier disruption, basement membrane degradation, brain swelling, and neuronal death [26]. These findings emphasize the significance of recognizing the island sign as a prognostic indicator in ICH, underscoring its potential utility in guiding treatment strategies and enhancing patient outcomes. Specifically, the "swirl sign" emerged as an independent predictor of an unfavorable prognosis at 3 months post-ICH as well as at 12 months.

The swirl sign, identified by Selariu E in 2017 [22], has emerged as a significant predictor of one-month mortality and poor functional outcomes at three months. However, conflicting results were reported by Kim et al. [27], suggesting that although the swirl sign is associated with higher mortality rates, it does not independently forecast outcomes. This radiological sign presents as areas of low or equal density within regions of high density relative to the brain parenchyma, indicating ongoing hemorrhage in the acute phase. The persistence of hemorrhage may indicate a more unfavorable prognosis. Despite some inconsistencies in its predictive capacity, detecting the swirl sign on imaging remains crucial as it offers insights into the evolving nature of hemorrhage progression and assists in assessing the risk of adverse outcomes in patients with ICH. Further investigation into its prognostic implications and underlying mechanisms is necessary to enhance its clinical usefulness.

Additionally, factors such as increasing age, intraventricular hemorrhage, and HE were linked to worse ICH prognoses, consistent with prior research [28–30]. While early imaging indicators, such as heterogeneous density and irregular hematoma shape, can predict HE, further exploration is needed to determine their specific implications for ICH prognosis. These findings from the long follow up results highlight the crucial role of early NCCT imaging in prognosis assessment and stress the importance of comprehensive evaluation.

Barras and colleagues previously utilized five categories to evaluate hematoma density and shape: homogeneous/regular (Category 1 to 2) or heterogeneous/irregular (Category 3 to 5) [31]. Delcourt et al. identified that an irregular shape, rather than heterogeneous density, was independently associated with unfavorable outcomes at 90 days post-ICH [32]. This correlation may be attributed to irregularly shaped hematomas triggering prolonged inflammation surrounding the lesion [33]. Alternatively, irregularities could worsen edema around the hematoma, a recognized predictor of poor prognosis [25]. The presence of hematoma density heterogeneity may theoretically indicate an adverse prognosis. Variations in density are linked to thrombus formation and the accumulation of cellular components in the plasma [32]. Furthermore, the heterogeneous pattern may indicate ongoing or recurrent bleeding, manifesting as a blend of fresh blood and older hematoma [25]. A comprehensive understanding of these nuances in hematoma characteristics is imperative for prognostic evaluation and treatment strategizing in ICH patients.

Studies have shown that the detection of satellite signs on the initial NCCT scan within 12 h is indicative of poor functional outcomes upon discharge for individuals with ICH [20, 34]. Recent researches suggests that hypodensities and black hole signs may serve as prognostic indicators for unfavorable outcomes in patients with ICH [13, 35–37]. The blend sign, defined by the coexistence of hypoattenuated and hyperattenuated areas within the hematoma with a density discrepancy of at least 18 HU between the 2 density regions [23], has been linked to an increased risk of impairment at three months based on CT scan results [23, 38]. The previous studies suggest these NCCT signs have the potential to advance our comprehension of ICH dynamics and guide clinical management strategies. Our study showed that the model with NCCT signs has a precise prognosis prediction value, which agrees with previous studies [22–38].

MRS score is used to evaluate the neurological recovery status and degree of disability in stroke patients [11, 39]. The lower is the score, the better recovery is the patient. The post-ICH mRS score significantly indicates the prognosis in this study, as reported in previous studies [11, 39, 40].

### Limitations

Our study has several limitations. Firstly, it was a retrospective cohort study conducted at a single institution, potentially introducing selection bias by including only patients who underwent an initial NCCT scan within 24 h of symptom onset and completed follow-up. Secondly, the study occurred at a large general hospital in a central local area, possibly resulting in poorer clinical conditions among admitted patients. Thirdly, the imcomplete data on HE, and the lack of the analysis on initial intracerebral hemorrhage volume, limiting a comprehensive understanding of the relationship between NCCT findings and prognosis. Fourthly, the direct segmentation method can provide more accurate results, but it also requires higher image quality. However, this retrospective study provided early CT scan results which are not of high quality, thus, a rough calculation method of ABC/2 was chosen for this study analysis. In case of in case of heterogeneous shape, the ABC/2 calculation may lead to some bias to the results. Lastly, the predictive model included only common clinical factors and NCCT markers, but not considering of the known factors of poor prognosis. This indicates the necessity for further optimization by incorporating new and robust predictive factors that may arise in future research.

## Conclusion

In conclusion of this long follow-up study, early NCCT imaging markers are crucial in prognostic assessment for patients with ICH. The incorporation of NCCT findings within 24 h significantly improves prognostic accuracy. Our novel integrated modeling approach, combining biochemical characteristics and NCCT results, facilitates precise prognosis prediction based on diverse features. These models support healthcare professionals in patient monitoring and optimizing treatment strategies. While logistic regression models consistently exhibit robust performance, ongoing calibration refinement is essential. Further research is warranted to validate prognostic predictions in larger patient cohorts and investigate how integrating multiple features can enhance the predictive capacity for the intensive management of ICH.

## Supplementary Information

Supplementary Material 1.

Supplementary Material 2.

## Data Availability

The datasets used and/or analysed during the current study are available from the corresponding author on reasonable request.

## Abbreviations

ICH: Intracerebral hemorrhage
HE: Hematoma expansion
CT: Computed tomography
mRs: Modified Rankin Scale
GCS: Glasgow Coma Scale
NIHSS: National Institutes of Health Stroke Scale
TC: Total Cholesterol
TG: Triglycerides
HDL: High-density Lipoprotein
LDL: Low-density Lipoprotein
CRP: C-reactive protein
CR: Creatinine
GFR: Glomerular Filtration Rate
PLT: Platelet count
ALT: Alanine Aminotransferase
AST: Aspartate Aminotransferase
GHB: Glycated Hemoglobin
PT-INR: Prothrombin Time International Normalized Ratio
FIB: Fibrinogen
HU: Hounsfield units
IQR: Interquartile range
ORs: Odds ratios
CIs: Confidence intervals
ROC: Receiver operating characteristic
AUC: Area under the ROC curve
NRI: Net reclassification index
IDI: Integrated discrimination improvement
