# Supplementary material for: Early NCCT imaging signs for prognostication in intracerebral hemorrhage: a retrospective cohort study with long follow up results
Source: BMC Neurol. 2025 Mar 6;25:91. doi: 10.1186/s12883-025-04100-z (PMC11883969; doi:10.1186/s12883-025-04100-z)
Supplement: Supplementary file 1 — Supplementary Material 1. [file 12883_2025_4100_MOESM1_ESM.docx]

Supplemental Table 1. The Comparison of characteristic data between the poor and good prognosis groups at 3 months post-onset

| Characteristic data | Variables | | Poor prognosis  （N=214）  N(%)/Mean±SD/Median(IQR) | Good prognosis  （N=237）  N(%)/Mean±SD/Median(IQR) | P | OR/ T index/ U index | 95% CI |
| --- | --- | --- | --- | --- | --- | --- | --- |
| Demographic | Age, years | | 62.28±11.95 | 56.50±11.55 | **<0.001** | 1.043 | 1.026,1.060 |
|  | Gender | Male | 123(57.5) | 157(66.2) | 0.055 | 1.452 | 0.991,2.127 |
|  |  | Female | 91(42.5) | 80(33.8) |  |  |  |
|  | Treatment modality | Only Medical | 88(41.1) | 140(59.1) | **<0.001** | 2.067 | 1.419,3.009 |
|  |  | MIS+ Medical | 126(58.9) | 97(40.9) |  |  |  |
| Past History | Hypertension | 0 | 56(26.2) | 46(19.4) | 0.087 | 0.680 | 0.436,1.059 |
|  |  | 1 | 158(73.8) | 191(80.6) |  |  |  |
|  | Diabetes | 0 | 200(93.5) | 210(88.6) | 0.074 | 0.544 | 0.278,1.068 |
|  |  | 1 | 14(6.5) | 27(11.4) |  |  |  |
|  | Coronary Heart Disease | 0 | 202(94.4) | 226(95.4) | 0.641 | 1.221 | 0.527,2.827 |
|  |  | 1 | 12(5.6) | 11(4.6) |  |  |  |
|  | Atrial Fibrillation | 0 | 212(99.1) | 234(98.7) | 1.000 | 0.736 | 0.122,4.446 |
|  |  | 1 | 2(0.9) | 3(1.3) |  |  |  |
|  | Anticoagulant/Antiplate Drug Use | 0 | 203(94.9) | 233(98.3) | **0.041** | 3.156 | 0.990,10.066 |
|  |  | 1 | 11(5.1) | 4(1.7) |  |  |  |
|  | Previous Stroke | 0 | 178(83.2) | 196(82.7) | 0.893 | 0.967 | 0.591,1.581 |
|  |  | 1 | 36(16.8) | 41(17.3) |  |  |  |
|  | Smoking | 0 | 138(64.5) | 154(65.0) | 0.913 | 1.022 | 0.694,1.504 |
|  |  | 1 | 76(35.5) | 83(35.0) |  |  |  |
|  | Alcohol Consumption | 0 | 142(66.4) | 145(61.2) | 0.254 | 0.799 | 0.543,1.175 |
|  |  | 1 | 72(33.6) | 92(38.8) |  |  |  |
| Admission data | Systolic blood pressure, mmHg | | 166.07±26.60 | 161.07±24.30 | 0.052 | 1.941 |  |
|  | Diastolic blood pressure, mmHg | | 93.75±16.27 | 94.10±18.41 | 0.812 | 0.238 |  |
|  | SMASH-U Classification | Structural lessons | 8(3.7) | 20(8.4) | 0.134 | 1.499 | 0.451,2.068 |
|  |  | Medication | 7(3.3) | 5(2.1) |  |  |  |
|  |  | Amyloid angiopathy | 19(8.9) | 17(7.2) |  |  |  |
|  |  | Systemic disease | 6(2.8) | 10(4.2) |  |  |  |
|  |  | Hypertension | 174(81.3) | 185(78.1) |  |  |  |
|  |  | Undetermined | 0(0.0) | 0(0.0) |  |  |  |
| Admission score | mRS before ICH | | 0(1) | 0(0) | **0.009** | 2.602 |  |
|  | mRS after ICH | | 5(1) | 4(2) | **<0.001** | 5.380 |  |
|  | GCS | | 12(7) | 14(4) | **<0.001** | -4.568 |  |
|  | NIHSS | | 16(11) | 12(12) | **<0.001** | 5.209 |  |
| CT imaging data | Hematoma location | Lobar | 52(24.3) | 58(24.5) | 0.815 | 0.233 | 0.045,0.451 |
|  |  | Deep | 138(64.5) | 155(65.4) |  |  |  |
|  |  | Cerebellar | 8(3.7) | 10(4.2) |  |  |  |
|  |  | Brainstem | 16(7.5) | 14(5.9) |  |  |  |
|  | Hematoma expansion* | 0 | 134(76.1) | 170(89.5) | **0.001** | 2.664 | 1.494,4.752 |
|  |  | 1 | 42(23.9) | 20(10.5) |  |  |  |
|  | Hematoma volume, ml | | 26.62±22.57 | 19.34±16.67 | **0.001** | 3.459 |  |
|  | Time from CT to onset, hours | | 2.86±3.07 | 4.19±3.50 | **0.000** | -3.800 |  |
|  | Ventricular hemorrhage | 0 | 114(53.3) | 159(67.1) | **0.003** | 1.788 | 1.221,2.619 |
|  |  | 1 | 100(46.7) | 78(32.9) |  |  |  |
|  | Subarachnoid hemorrhage | 0 | 166(77.6) | 189(79.7) | 0.573 | 1.139 | 0.725,1.788 |
|  |  | 1 | 48(22.4) | 48(20.3) |  |  |  |
|  | Midline shift | 0 | 42(19.6) | 33(13.9) | 0.104 | 0.662 | 0.402,1.091 |
|  |  | 1 | 172(80.4) | 204(86.1) |  |  |  |
|  | Midline shift direction | Left | 96(55.8) | 108(52.9) | 0.577 | 0.891 | 0.592,1.339 |
|  |  | Right | 76(44.2) | 96(47.1) |  |  |  |
| Serological indicators | Total Cholesterol, TC, mmol/L | | 4.39±0.88 | 4.42±0.97 | 0.875 | -0.157 |  |
|  | Triglyceride, TG, mmol/L | | 1.44±0.84 | 1.71±2.26 | 0.623 | -0.492 |  |
|  | High density lipoprotein, HDL, mmol/L | | 1.29±0.40 | 1.23±0.37 | 0.200 | 1.281 |  |
|  | Low-density lipoprotein, LDL, mmol/L | | 2.72±0.79 | 2.79±0.85 | 0.720 | -0.358 |  |
|  | C-reactive Protein, CRP, mg/L | | 13.30±30.45 | 16.49±36.88 | 0.369 | -0.898 |  |
|  | Creatinine, CR, umol/L | | 78.46±32.78 | 93.01±142.73 | 0.460 | 0.738 |  |
|  | Glomerular Filtration Rate, GFR, mL/min | | 83.17±26.91 | 88.66±32.76 | **0.041** | -2.045 |  |
|  | Platelet, PLT, *10^9/L | | 206.59±63.93 | 209.71±63.25 | 0.521 | -0.642 |  |
|  | Alanine Aminotransferase, ALT (U/L) | | 23.06±22.27 | 20.69±18.47 | 0.183 | 1.331 |  |
|  | Aspertate Aminotransferase, AST (U/L) | | 27.80±15.98 | 24.97±14.49 | **0.005** | 2.799 |  |
|  | Glycated Hemoglobin, GHB, mmol/L | | 5.85±1.13 | 5.97±1.04 | 0.069 | -1.821 |  |
|  | Prothrombin Time International Normalized Ratio, PT-INR | | 1.02±0.22 | 1.00±0.11 | 0.636 | -0.474 |  |
|  | Fibrinogen, FIB, g/L | | 3.37±1.16 | 3.57±1.23 | 0.073 | -1.791 |  |

A good prognosis was defined as an mRS score lower than 3, whereas a poor prognosis, including death (score of 6), was indicated by a score of 3 or higher.
