# Supplementary material for: Early NCCT imaging signs for prognostication in intracerebral hemorrhage: a retrospective cohort study with long follow up results
Source: BMC Neurol. 2025 Mar 6;25:91. doi: 10.1186/s12883-025-04100-z (PMC11883969; doi:10.1186/s12883-025-04100-z)
Supplement: Supplementary file 2 — Supplementary Material 2. [file 12883_2025_4100_MOESM2_ESM.docx]

Supplemental Table 2. The Comparison of characteristic data between the poor and good prognosis groups at 12 months post-onset

| Characteristic data | Variables | | Poor prognosis  （N=199）  N(%)/Mean±SD/Median(IQR) | Good prognosis  （N=252）  N(%)/Mean±SD/Median(IQR) | P | OR | 95% CI |
| --- | --- | --- | --- | --- | --- | --- | --- |
| Demographic | Age, years | | 62.40±11.87 | 56.75±11.68 | **<0.001** | 5.036 |  |
|  | Gender | Male | 112(56.3) | 168(66.7) | **0.024** | 1.554 | 1.059,2.280 |
|  |  | Female | 87(43.7) | 84(33.3) |  |  |  |
|  | Treatment modality | Only Medical | 77(38.7) | 151(59.9) | **<0.001** | 2.369 | 1.619,3.466 |
|  |  | MIS+ Medical | 122(61.3) | 101(40.1) |  |  |  |
| Past History | Hypertension | 0 | 53(26.6) | 49(19.4) | 0.070 | 0.665 | 0.427,1.035 |
|  |  | 1 | 146(73.4) | 203(80.6) |  |  |  |
|  | Diabetes | 0 | 186(93.5) | 224(88.9) | 0.093 | 0.559 | 0.282,1.110 |
|  |  | 1 | 13(6.5) | 28(11.1) |  |  |  |
|  | Coronary Heart Disease | 0 | 188(94.5) | 240(95.2) | 0.714 | 1.170 | 0.505,2.711 |
|  |  | 1 | 11(5.5) | 12(4.8) |  |  |  |
|  | Atrial Fibrillation | 0 | 198(99.5) | 248(98.4) | 0.522 | 0.313 | 0.035,2.824 |
|  |  | 1 | 1(0.5) | 4(1.6) |  |  |  |
|  | Anticoagulant/Antiplate Drug Use | 0 | 190(95.5) | 246(97.6) | 0.208 | 1.942 | 0.679,5.551 |
|  |  | 1 | 9(4.5) | 6(2.4) |  |  |  |
|  | Previous Stroke | 0 | 166(83.4) | 208(82.5) | 0.806 | 0.940 | 0.573,1.542 |
|  |  | 1 | 33(16.6) | 44(17.5) |  |  |  |
|  | Smoking | 0 | 132(66.3) | 160(63.5) | 0.531 | 0.883 | 0.598,1.304 |
|  |  | 1 | 67(33.7) | 92(36.5) |  |  |  |
|  | Alcohol Consumption | 0 | 137(68.8) | 150(59.5) | **0.041** | 0.666 | 0.450,0.984 |
|  |  | 1 | 62(31.2) | 102(40.5) |  |  |  |
| Admission data | Systolic blood pressure, mmHg | | 165.87±27.79 | 161.52±23.44 | 0.195 | 1.296 |  |
|  | Diastolic blood pressure, mmHg | | 93.38±16.01 | 94.38±18.46 | 0.823 | -0.223 |  |
|  | SMASH-U Classification | Structural lessons | 5(2.5) | 23(9.1) | 0.013 | 2.477 | 1.451,3.110 |
|  |  | Medication | 9(4.5) | 3(1.2) |  |  |  |
|  |  | Amyloid angiopathy | 17(8.5) | 19(7.5) |  |  |  |
|  |  | Systemic disease | 6(3.0) | 10(4.0) |  |  |  |
|  |  | Hypertension | 162(81.5) | 197(78.2) |  |  |  |
|  |  | Undetermined | 0(0.0) | 0(0.0) |  |  |  |
| Admission score | mRS before ICH | | 0(1) | 0(0) | **0.003** | 2.925 |  |
|  | mRS after ICH | | 5(1) | 4(2) | **<0.001** | 5.500 |  |
|  | GCS | | 12(6) | 14(4) | **<0.001** | -5.028 |  |
|  | NIHSS | | 16(10) | 12(12) | **<0.001** | 5.339 |  |
| CT imaging data | Hematoma location | Lobar | 45(22.6) | 65(25.8) | 0.444 | 0.765 | 0.035,1.009 |
|  |  | Deep | 132(66.3) | 161(63.9) |  |  |  |
|  |  | Cerebellar | 7(3.6) | 11(4.3) |  |  |  |
|  |  | Brainstem | 15(7.5) | 15(6.0) |  |  |  |
|  | Hematoma expansion* | 0 | 121(74.2) | 183(90.1) | **<0.001** | 3.176 | 1.778,5.672 |
|  |  | 1 | 42(25.8) | 20(9.9) |  |  |  |
|  | Hematoma volume, ml | | 26.47±22.71 | 19.90±17.07 | **0.003** | 3.001 |  |
|  | Time from CT to onset, hours | | 2.89±3.14 | 4.03±3.45 | **<0.001** | -3.901 |  |
|  | Ventricular hemorrhage | 0 | 106(53.3) | 167(66.3) | **0.005** | 1.724 | 1.177,2.525 |
|  |  | 1 | 93(46.7) | 85(33.7) |  |  |  |
|  | Subarachnoid hemorrhage | 0 | 154(77.4) | 201(79.8) | 0.541 | 1.152 | 0.732,1.811 |
|  |  | 1 | 45(22.6) | 51(20.2) |  |  |  |
|  | Midline shift | 0 | 39(19.6) | 36(14.3) | 0.132 | 0.684 | 0.416,1.124 |
|  |  | 1 | 160(80.4) | 216(85.7) |  |  |  |
|  | Midline shift direction | Left | 90(56.3) | 114(52.8) | 0.504 | 0.869 | 0.576,1.311 |
|  |  | Right | 70(43.7) | 102(47.2) |  |  |  |
| Serological indicators | Total Cholesterol, TC, mmol/L | | 4.38±0.89 | 4.43±0.95 | 0.765 | -0.299 |  |
|  | Triglyceride, TG, mmol/L | | 1.48±0.87 | 1.66±2.18 | 0.840 | 0.202 |  |
|  | High density lipoprotein, HDL, mmol/L | | 1.27±0.38 | 1.26±0.39 | 0.710 | 0.372 |  |
|  | Low-density lipoprotein, LDL, mmol/L | | 2.72±0.81 | 2.78±0.84 | 0.674 | -0.421 |  |
|  | C-reactive Protein, CRP, mg/L | | 14.76±32.09 | 15.18±35.47 | 0.827 | -0.219 |  |
|  | Creatinine, CR, umol/L | | 77.73±30.70 | 92.63±138.66 | 0.472 | 0.720 |  |
|  | Glomerular Filtration Rate, GFR, mL/min | | 81.72±26.28 | 89.42±32.62 | **0.002** | -3.055 |  |
|  | Platelet, PLT, *10^9/L | | 206.17±63.14 | 209.83±63.90 | 0.556 | -0.589 |  |
|  | Alanine Aminotransferase, ALT (U/L) | | 23.21±22.90 | 20.73±18.13 | 0.184 | 1.328 |  |
|  | Aspertate Aminotransferase, AST (U/L) | | 28.43±17.03 | 24.65±13.54 | **0.003** | 2.979 |  |
|  | Glycated Hemoglobin, GHB, mmol/L | | 5.84±1.16 | 5.97±1.02 | **0.018** | -2.358 |  |
|  | Prothrombin Time International Normalized Ratio, PT-INR | | 1.01±0.21 | 1.01±0.15 | 0.450 | -0.756 |  |
|  | Fibrinogen, FIB, g/L | | 3.37±1.18 | 3.56±1.21 | 0.080 | -1.749 |  |

A good prognosis was defined as an mRS score lower than 3, whereas a poor prognosis, including death (score of 6), was indicated by a score of 3 or higher.
